# Knowledge, Attitudes, and Practices of Hungarian General Practitioners Regarding Human Papillomavirus (HPV) Infection and Vaccination: A Nationwide Cross-Sectional Study

**DOI:** 10.3390/vaccines14020196

**Published:** 2026-02-22

**Authors:** Richárd Tóth, Pál Sebok, Eszter Börzsönyi, Icó Tóth, Barbara Sebők, Balázs Vida, Ferenc Bánhidy, Márton Keszthelyi, Balázs Lintner

**Affiliations:** 1 Department of Obstetrics and Gynecology, Semmelweis University, 1082 Budapest, Hungary; sebok.pal.szabolcs@semmelweis.hu (P.S.); borzsonyi.eszter@semmelweis.hu (E.B.); vida.balazs.lajos@semmelweis.hu (B.V.); banhidy.ferenc@semmelweis.hu (F.B.); keszthelyi.marton@semmelweis.hu (M.K.); lintner.balazs.zoltan@semmelweis.hu (B.L.); 2 Mallow Flower Foundation, 1111 Budapest, Hungary; toth.ico@malyvavirag.hu; 3 Workgroup of Research Management, Doctoral School, Semmelweis University, 1085 Budapest, Hungary; sebok.barbara@semmelweis.hu

**Keywords:** human papillomavirus, cervical cancer, vaccination, general practitioners, primary care

## Abstract

**Objective**: To evaluate the level of knowledge, attitudes, and practices of Hungarian general practitioners (GPs) concerning human papillomavirus (HPV) infection, cervical cancer prevention, and HPV vaccination, and to identify physician-level factors associated with proactive recommendation practices. **Methods**: A cross-sectional nationwide survey was conducted between 30 April and 1 June 2024. The online questionnaire was distributed to practicing Hungarian GPs listed in the National Health Insurance Fund database. Anonymous responses were collected on demographic data, knowledge of HPV transmission and oncogenic potential, awareness of vaccination guidelines, and clinical counseling habits. Descriptive and inferential statistical analyses were performed. A total of 413 responses were received. **Results**: Most respondents were female (72.6%) with an average of 22.4 ± 9.6 years of professional experience. Although 89.8% correctly identified the causal link between HPV and cervical cancer, only 56.2% were aware of the complete vaccination schedule recommended for adolescents initiating after age 15. Knowledge scores were significantly higher among female physicians, urban practitioners, and those with postgraduate preventive medicine training. While the overall attitude toward HPV vaccination was positive (mean 4.6/5), 38.4% of respondents reported parental hesitancy as a common barrier, often citing misinformation regarding vaccine safety (64.9%) and lack of perceived need for boys (58.7%). Regression analysis revealed that familiarity with WHO and national vaccination guidelines independently predicted proactive vaccine recommendation (β = 0.43, *p* < 0.001). **Conclusions**: Hungarian general practitioners demonstrate good baseline awareness of HPV and its oncogenic role; however, knowledge gaps persist regarding vaccination schedules and counseling practices. Enhancing continuous medical education and communication training could strengthen GPs’ role as key advocates in HPV vaccine promotion.

## Simple Summary

This nationwide survey assessed Hungarian general practitioners’ (GPs) knowledge, attitudes, and practices regarding HPV infection, and HPV vaccination. Between 30 April and 1 June 2024, an online questionnaire collected demographic data, knowledge of HPV transmission, awareness of vaccination guidelines, and counseling behaviors. A total of 413 GPs responded, most of whom were female with over two decades of experience. While general awareness of HPV’s causal role in cervical cancer was high, many respondents lacked full knowledge of the recommended vaccination schedule for adolescents starting after age 15. Knowledge levels were higher among female physicians, urban practitioners, and those with preventive medicine training. Although attitudes toward HPV vaccination were strongly positive, parental hesitancy—driven by misinformation and low perceived need for vaccinating boys—remained a frequent barrier. Familiarity with WHO and national guidelines strongly predicted proactive vaccine recommendation. Strengthened professional education and communication skills could enhance GPs’ role in improving vaccination uptake.

## 1. Introduction

Despite being one of the most preventable malignancies, cervical cancer continues to present a significant global health burden. With more than 650,000 new cervical cancer cases and approximately 350,000 deaths annually, it is the fourth most common cancer among women [1]. In Hungary, cervical cancer accounts for nearly 1000 new diagnoses and 500 deaths per year, ranking sixth among female malignancies and third among women younger than 55 years [1]. Persistent infection with high-risk human papillomavirus (HPV) types—particularly HPV 16 and 18—is responsible for over 95% of these cases [2].

In recent decades, the implementation of cervical screening programs and HPV vaccination campaigns dramatically reduced cervical cancer incidence in many high-income countries [3,4,5]. Although HPV vaccination has been incorporated into national immunization strategies across Europe, variation in uptake persists, indicating that implementation factors—particularly physician recommendation practices—may play a critical role. Understanding these determinants within primary care settings is therefore relevant beyond individual national contexts [6,7].

The World Health Organization’s (WHO) Global Strategy to Eliminate Cervical Cancer sets a “90–70–90” target: 90% of girls fully vaccinated against HPV by age 15, 70% of women screened at least once by age 35 and again by age 45, 90% of women with cervical disease receiving appropriate treatment [8].

Nevertheless, HPV vaccination programs across Europe show substantial variation in implementation and coverage. Recent European data indicate that full vaccination coverage ranges widely, from approximately 92% in Portugal to around 9% in Bulgaria among females and 91% in Portugal to 7% in Poland among males (Supplementary Table S1) [9,10,11,12]. In most European countries, HPV vaccination is now offered to both girls and boys, reflecting a shift toward gender-neutral prevention strategies, although coverage among boys generally remains lower due to their more recent inclusion in vaccination programs [10,11,12,13]. Target ages for routine vaccination are typically in early adolescence (approximately 9–14 years), although national schedules differ in timing and delivery settings. This heterogeneity suggests that system-level availability alone does not determine uptake and highlights the importance of healthcare provider recommendation practices [10,11].

In Hungary, HPV vaccination implemented as part of the National Immunization Program as a school-based campaign. The vaccine is offered voluntarily and free of charge to students in the 7th grade (typically 12–13 years of age). The program initially targeted girls (introduced nationally in the 2014/2015 school year) and was later expanded to include boys (from the 2020/2021 school year). Parents receive standardized information materials and consent forms through schools as part of national public health communication campaigns. Outside the organized school-based program, HPV vaccination is generally available only through self-financed purchase with prescription [14].

It can be seen that achieving the ambitious WHO goals requires action on several levels, and the involvement of numerous medical and non-medical participants to build trust and to enhance acceptance of vaccination [7]. Among doctors, primary care physicians play a critical role in promoting preventive measures. General practitioners (GPs) and family pediatricians often serve as the first point of contact for families and adolescents, providing trusted advice and counseling about vaccination. Numerous studies confirm that physician recommendation is one of the strongest predictors of HPV vaccine uptake [15,16,17].

Conversely, lack of knowledge, uncertainty about safety, limited communication skills, and time constraints can hinder vaccine advocacy among primary care providers [18,19].

As aforementioned, Hungary has implemented a structured, school-based HPV vaccination program, several implementation-related questions remain unresolved. Vaccination is offered voluntarily and free of charge to adolescents within the national immunization program, and parental consent is required for participation. Among uncertain parents, physician communication and counselling—particularly by general practitioners and family pediatricians—may substantially influence parental attitudes, vaccine acceptance, and program success [19,20,21,22]. Although attitudes toward HPV vaccination of adults and adolescents were assessed in Hungary, no nationwide study has previously examined the knowledge, attitudes, and counseling practices of Hungarian primary care physicians regarding HPV vaccination [22,23]. Understanding these physician-level factors is therefore essential to identify potential barriers within an otherwise organized prevention framework and to inform strategies aimed at improving vaccination uptake [24,25,26].

The present study aimed to fill this knowledge gap by conducting a nationwide survey among Hungarian general practitioners. The objectives were:

- To evaluate GPs’ knowledge of HPV infection, transmission, and oncogenic potential.
- To assess attitudes toward HPV vaccination and perceived barriers to vaccine recommendation.
- To identify sociodemographic and professional predictors of proactive vaccination behavior.

Ultimately, this study intends to support the development of focused educational programs and reinforce primary care’s role in meeting Hungary’s HPV vaccination objectives.

## 2. Materials and Methods

### 2.1. Study Design and Participants

A nationwide, cross-sectional survey was conducted between 30 April and 1 June 2024, targeting practicing Hungarian general practitioners (GPs), including adult, pediatric, and mixed practice types, as HPV vaccination counselling in Hungary is not restricted exclusively to pediatric settings. The sampling frame was derived from the Hungarian National Health Insurance Fund Administration (NEAK) public database, which contains official information of all licensed GPs in Hungary.

A minimum required sample size was estimated for descriptive analyses using the national number of practicing general practitioners and family pediatricians in Hungary (*N* = 5469). Assuming a 95% confidence level and a 5% margin of error, the required sample size was approximately 360 respondents. The final sample (*n* = 413) therefore provided adequate precision for the planned analyses.

Using publicly accessible professional contact information, valid email addresses could be identified for 2417 physicians, who were invited to participate in the survey.

A total of 2417 physicians were invited to participate through an anonymous online survey. The invitation letter briefly described the study aims, confidentiality, and voluntary nature of participation. Informed consent was obtained electronically from all participants prior to participation. Completion of the anonymous questionnaire was considered as implied consent.

Responses were accepted from all counties and Budapest, ensuring national coverage. After data cleaning, 413 fully completed responses were included in the analysis, yielding a response rate of 17.1%. Respondents were categorized into adult (*n* = 183, 44.3%), pediatric (*n* = 142, 34.4%), and mixed (*n* = 88, 21.3%) practice types. For analytical purposes, mixed practices were combined with adult practices due to their predominant provide adult care.

### 2.2. Questionnaire Development

The research instrument consisted of a core and an ancillary questionnaire, of which the latter was provided in three formats tailored to each practice type (adult, pediatric, mixed) to ensure clinical relevance. The questionnaires were designed collaboratively with the Mallow flower Foundation, a Hungarian non-governmental organization (NGO) active in HPV awareness and gynecologic oncology patient advocacy, to ensure the representation of patients’ perspective in the survey. Each version contained 47–50 items divided into four sections:

- Demographic and professional background:

Gender, age, years in practice, type and size of patient population, and regional setting (urban, semi-urban, rural).

- Knowledge about HPV infection and transmission:

Modes of transmission, oncogenic types, relationship with cervical and other cancers, and knowledge of screening recommendations.

- Knowledge and attitudes toward HPV vaccination:

Vaccine composition, target age groups, number of doses, gender inclusion, contraindications, safety perceptions, and perceived efficacy.

- Facilitators and barriers to vaccine recommendation:

Perceived patient and parental attitudes, confidence in discussing sexually transmitted infections, and physicians’ comfort level in vaccine counseling.

Responses included multiple-choice questions, true/false statements, and Likert-scale items (1 = strongly disagree to 5 = strongly agree). Open-ended questions were included for qualitative insights. The estimated completion time was 6–8 min.

The questionnaire underwent pilot testing among 15 GPs to ensure clarity and internal consistency. Minor linguistic adjustments were made before nationwide dissemination.

Internal consistency analysis was performed on the numerically coded questionnaire items using complete-case data. After exclusion of responses with missing values across the included items, a total of 13 complete responses were available for reliability analysis, comprising 70 items.

The overall internal consistency of the questionnaire was excellent, with a Cronbach’s alpha coefficient of 0.996, indicating a very high level of inter-item correlation. Corrected item–total correlations ranged from 0.779 to 0.998, suggesting strong alignment of individual items with the overall scale.

Item-level diagnostics showed that removal of any single item did not substantially improve reliability; Cronbach’s alpha if item deleted ranged between 0.996 and 0.996, indicating that no individual item disproportionately reduced scale consistency.

These findings demonstrate a highly coherent response pattern across items; however, the extremely high alpha value may also indicate potential item redundancy and conceptual overlap among questionnaire domains.

Detailed questionnaire provided in the Supplementary Materials.

### 2.3. Data Collection

The survey was distributed via online platform (Google Forms, Google LLC, Mountain View, CA, USA). Responses were stored anonymously and automatically exported into IBM SPSS Statistics 28.0 (IBM Corp., Armonk, NY, USA) for analysis. IP tracking and identifying information were disabled to ensure respondent confidentiality.

### 2.4. Scoring System

To assess knowledge regarding HPV infection and prevention, we used a composite knowledge score (0–9 points) based on the number of correct answers across designated knowledge items. For multiple-choice questions, participants were instructed to select all applicable options. A score was awarded only when all correct answers were selected, and no incorrect answers were marked; partial credit was not applied. This approach reduced the influence of guessing and allowed quantification of participants’ overall knowledge level, facilitating statistical comparison across study groups. This allowed the quantification of participants’ overall knowledge level and facilitated statistical comparison.

Attitude toward vaccination was assessed using the mean of Likert-scale items evaluating trust in vaccine safety, efficacy, and importance in cancer prevention.

### 2.5. Statistical Analysis

Statistical analyses were performed to examine associations between demographic characteristics, HPV-related knowledge, attitudes toward HPV vaccination, and vaccination practices.

A composite HPV Knowledge Score (0–9) was calculated by summing correct responses to nine knowledge items (What type of infection is HPV?; Is genital HPV infection regularly associated with symptoms?; What diseases can be caused by HPV infection?; What percentage of cervical cancers is attributable to HPV infection?; Which HPV types can cause genital warts?; Which HPV types can cause cervical cancer?; In which age groups is newly acquired HPV infection most common?; Who should be screened for HPV infection?; How many components does the currently available HPV vaccine in Hungary contain?). For descriptive and subgroup analyses, the score was categorized into three levels: poor (0–3), fair (4–6), and excellent (7–9). The Knowledge Score was treated as a continuous variable.

Likert-scale items (self-rated HPV knowledge and perceived HPV vaccine effectiveness, 1–5) were analyzed as ordinal variables.

The following variables were handled as categorical (nominal) variables: sex, vaccination recommendation (yes/no), dichotomized perceived vaccine effectiveness (adequate vs. not adequate), dichotomized self-rated knowledge (good vs. poor), and the four respondent groups representing the source of questionnaire completion (adult GPs, pediatric GPs, conference participants, mixed group).

Original age categories (26–30, 31–40, 41–50, 51–60, 61–70, ≥70 years) and years in practice (0–4, 5–9, 10–19, 20–29, ≥30 years) were ordinal by definition; these were treated as ordinal variables in correlation analyses, and as categorical variables in χ^2^ tests. A derived two-level age grouping (≤50 vs. >50 years) was also used for comparative analyses.

Normality was assessed only for the continuous Knowledge Score using the Shapiro–Wilk test; as the distribution significantly deviated from normality, non-parametric methods were applied. Spearman’s rank correlation was used to assess associations between continuous or ordinal variables.

Associations between categorical variables were examined using Pearson’s χ^2^ test. For significant 2 × 2 contingency tables, odds ratios (ORs) with 95% confidence intervals were calculated to quantify effect sizes.

Descriptive statistics included medians, ranges, and—where appropriate—means and standard deviations for continuous or ordinal variables, and absolute and relative frequencies for categorical variables. All statistical tests were two-sided, with *p* < 0.05 considered statistically significant.

All analyses were performed using IBM SPSS Statistics for Windows, Version 25.0 (IBM Corp., Armonk, NY, USA).

### 2.6. Ethical Considerations

The study was approved by the Institutional Review Board at Semmelweis University (Budapest, Hungary). The decision number is SE-RKEB-32/2024, dated 6 March 2024.

## 3. Results

### 3.1. Demographic Characteristics

A total of 413 questionnaires were analyzed. Majority (72.6%) of the participants were females. The highest proportion of respondents (28%) belonged to the 51–60-year age group, closely followed by the 41–50-year group (27%).

The distribution of respondents by years of experience in primary care showed that each group of 10 years’ experience in primary care practice was represented equally.

Most of the participants (58.11%) worked in clearly adult practices. More than half of the participants (54%) worked in the capital. Table 1 summarizes the demographic and professional characteristics of respondents

### 3.2. General HPV Knowledge and Attitudes Towards HPV Infection

Most respondents correctly identified HPV as a sexually transmitted infection (*n* = 389, 94%). The majority perceived HPV infection as prevalent or highly prevalent, with 270 physicians (65%) rating it as markedly or extremely prevalent. Genital HPV infection was correctly identified as predominantly asymptomatic by 368 respondents (89%).

Knowledge of HPV-related diseases varied by condition. Nearly all participants showed association of HPV with cervical cancer (*n* = 408; 99%) and genital warts (*n* = 403; 98%). High proportions also correctly identified penile cancer (*n* = 324; 78%), anal cancer (*n* = 275; 67%), malignancies of the oral cavity (*n* = 336; 81%), and pharyngeal cancer (*n* = 282; 68%). In contrast, 82 respondents (20%) incorrectly identified endometrial cancer and 45 (11%) ovarian cancer as HPV-related.

Regarding genotype-specific knowledge, HPV types 6 and 11 were identified as causative agents of genital warts by 329 (80%) and 291 (70%) respondents, respectively. High-risk oncogenic genotypes HPV 16 and 18 were correctly identified by 320 (77%) and 328 (79%) respondents. However, 124 (30%) and 126 (31%) respondents incorrectly associated HPV 16 and 18 with genital warts, respectively.

Most respondents identified the 20–30-year age group as having the highest incidence of new HPV infections (*n* = 320; 77%). Adult women were most frequently selected as candidates for HPV screening (*n* = 315; 76%), followed by individuals belonging to risk groups (*n* = 281; 68%) and adult men (*n* = 215; 52%). Screening of adolescent girls and boys was selected by 168 (41%) and 127 (31%) respondents, respectively.

HPV-PCR testing was considered the most effective screening method by 284 respondents (69%), followed by cytological examination (Pap test) by 230 (56%). Only a minority selected visual inspection-based methods (*n* = 17, 4%) or biomarker testing (*n* = 30; 7%). Almost all physicians reported a need for further education in this field (*n* = 391; 95%) (Table 2).

### 3.3. Perceived Professional Role, Knowledge Adequacy, and Stigma Related to HPV and Sexually Transmitted Infections (STIs)

Self-perceived adequacy of HPV-related knowledge was moderate to high. On a five-point Likert scale, 166 respondents (40%) rated their knowledge as high or very high (scores 4–5), while 196 (47%) selected the middle category. Only 51 physicians (12%) rated their knowledge as low or very low. The mean self-rated knowledge score was 3.31, with a median of 3.

Most respondents perceived discussing sexually transmitted infections as part of their professional responsibility, with 233 physicians (57%) rating this responsibility as high or very high. Responsibility for discussing HPV was rated even higher, with 286 respondents (69%) selecting scores of 4 or 5. The mean scores for perceived responsibility were 3.74 for STIs and 3.97 for HPV.

STIs were perceived as highly stigmatizing, with 308 respondents (74%) rating stigma levels as high or very high (scores 4–5; mean 4.08, median 4). In contrast, HPV infection was perceived as less stigmatizing: 166 physicians (40%) rated HPV-related stigma as high or very high, while 150 (36%) selected moderate levels. The mean stigma score for HPV was 3.20, with a median of 3 (Table 3).

### 3.4. Communication Comfort and Barriers Related to Sexual Health and HPV

Self-reported comfort in communicating about sexuality and HPV-related topics varied considerably by subject and patient group (Table 4). Communication about sexuality with adolescent boys was rated as comfortable or very comfortable (scores 4–5) by 64 respondents (37%), while 53 respondents (31%) reported low comfort levels (scores 1–2). The mean comfort score for this item was 2.19, with a median of 2. Communication about sexuality with adolescent girls yielded similar results, with a mean score of 1.91 and a median of 2; 81 respondents (47%) rated their comfort as high or very high, whereas 41 (24%) reported low comfort.

Discussing sexuality with parents was associated with moderate discomfort. Only 81 respondents (47%) reported high or very high comfort levels, while 37 (22%) indicated low comfort. Communication about sexuality with pediatric patients in the presence of parents showed a mean score of 2.06 and a median of 2, with 70 respondents (41%) reporting high comfort and 51 (29%) reporting low comfort.

Comfort levels differed markedly when communicating with adult patients. Talking about sexuality with adult men was rated as comfortable or very comfortable by 146 respondents (53%), with a mean score of 1.99 and a median of 2. Communication with adult women showed higher comfort levels, with 178 respondents (64%) reporting high or very high comfort (mean 2.39, median 2). This difference was observed in a sample predominantly composed of female physicians (72.6%).

Communication with non-heterosexual patients showed the greatest variability. High or very high comfort was reported by 128 respondents (46%), while 69 (25%) reported low comfort. The mean score for this item was 3.34, with a median of 3.

Confidence in discussing HPV specifically was higher than for general sexuality-related topics. A total of 279 respondents (67%) rated their confidence as high or very high, while only 32 (8%) reported low confidence. The mean confidence score for HPV-related communication was 2.48, with a median of 2.

Perceived barriers influencing the frequency of HPV vaccination recommendation are summarized in Table 5. Discomfort in communicating about sexuality was rated as not influential or minimally influential (scores 1–2) by 253 respondents (62%), with a mean score of 2.19 and a median of 2. Similarly, discomfort in communicating about HPV was rated as minimally influential by 296 respondents (72%) (mean 1.91, median 2).

Frequent changes in vaccination guidance and lack of data regarding vaccine efficacy or safety were also rated as minor barriers, with median scores of 2 for all three items. In contrast, lack of time in everyday practice emerged as the most prominent barrier. For this item, 194 respondents (45%) rated its influence as high or very high (scores 4–5), resulting in the highest mean score among all barriers (mean 3.34, median 3).

Perceived insufficiency of personal knowledge to responsibly initiate discussions about HPV was rated as a moderate barrier, with 117 respondents (28%) assigning high or very high influence scores and a mean score of 2.48 (median 2).

### 3.5. Physicians Perceptions of Patients’ and Parents’ Attitudes Toward HPV Vaccination

Physicians’ perceptions of patient’s attitudes toward HPV vaccination are summarized in Table 6. Most respondents perceived patients as ambivalent rather than overtly negative toward HPV vaccination. When asked whether patients have a negative attitude toward HPV vaccination, 53 respondents (19%) reported not at all, while 126 (45%) selected the middle category. The mean score for this item was 2.51, with a median of 3.

Discomfort related to discussing sexuality was perceived as common among patients. A total of 127 respondents (46%) rated patient discomfort as high or very high (scores 4–5), resulting in a mean score of 3.37 and a median of 3. Lack of interest in non-obligatory vaccinations was also frequently perceived, with 100 respondents (36%) rating this as moderately present and 100 (36%) rating it as highly or very highly present (mean 3.10, median 3).

Regarding vaccine-related beliefs, 124 respondents (45%) indicated that patients moderately doubted the efficacy of HPV vaccination, while 44 (16%) rated this doubt as high or very high (mean 2.70, median 3). Similar patterns were observed for perceived concerns about vaccine safety (mean 2.75, median 3). Underestimation of personal risk was frequently perceived, with 129 respondents (47%) rating this factor as high or very high (mean 3.36, median 3).

Cost-related concerns showed a distinct pattern. HPV vaccination being perceived as too expensive by patients was rated as highly or very highly influential by 208 respondents (76%), yielding the highest mean score among patient-related barriers (mean 4.18, median 5).

Physicians also reported mixed perceptions regarding patient’s self-assessed level of knowledge. While 203 respondents (73%) rated patient’s perceived knowledge about HPV as low or very low (scores 1–2), the perceived need for further information was rated as high or very high by 148 respondents (54%) (mean 3.56, median 4).

Perceptions of parental attitudes toward HPV vaccination are presented in Table 6. Parents were most commonly perceived as uncertain rather than strongly opposed to HPV vaccination. Negative parental attitudes toward vaccinating their children were rated as low by 109 respondents (63%), with a mean score of 2.31 and a median of 2.

Discomfort in discussing children’s sexual activity was perceived as common among parents, with 66 respondents (38%) rating this discomfort as high or very high (mean 3.15, median 3). Parents were generally perceived as not being well informed about their children’s sexuality, with 106 respondents (62%) rating parental awareness as low or very low (mean 2.32, median 2).

Parental communication regarding sexuality was most commonly rated at low to moderate levels (Table 6). A total of 92 respondents (53%) rated discussions about sexuality at home as infrequent (scores 1–2), while 70 (40%) selected the middle category. Only 11 respondents (6%) reported frequent discussions (scores 4–5), resulting in a mean score of 2.43 and a median of 2.

Cost-related concerns were again prominent. Vaccination being considered too expensive outside the national immunization program was rated as highly influential by 88 respondents (51%), with a mean score of 3.46 and a median of 4. In addition, 67 respondents (39%) rated concerns about excessive vaccination burden as high or very high (mean 3.15, median 3).

Finally, parents were perceived as having limited confidence in HPV vaccination, with 71 respondents (41%) rating parental doubts regarding vaccine efficacy as moderate and 19 (11%) as high or very high. Similar distributions were observed for perceived doubts about vaccine safety (mean 2.57, median 3). More than half of respondents (104, 56%) indicated that parents would benefit from additional information about HPV (mean 3.39, median 3).

### 3.6. Frequency and Initiation of HPV-Related Discussions in Clinical Practice

The frequency of sexual health–related topics discussed during routine consultations is summarized in Table 7. Discussions about condom use were reported to occur infrequently. A total of 285 respondents (69%) indicated that they discuss condom use rarely or very rarely (scores 1–2), while 92 (22%) selected the middle category. Only 36 respondents (9%) reported frequent discussions (scores 4–5). The mean score for this item was 2.22, with a median of 2.

Similarly, discussions about sexually transmitted infections were reported as infrequent. A total of 103 respondents (60%) rated STI-related discussions as rare or very rare, 52 (30%) selected the middle category, and only 6 respondents (3%) reported frequent discussions. The mean score was 2.23, with a median of 2.

HPV-related topics were discussed somewhat more frequently. When asked how often they discuss HPV and HPV-related pathologies, 89 respondents (52%) reported rare or very rare discussions, 72 (42%) selected the middle category, and 12 respondents (7%) reported frequent discussions. The mean score for this item was 2.51, with a median of 2.

Initiation of HPV-related discussions was most often physician-driven. A total of 105 respondents (61%) reported that they rarely or very rarely open the topic of HPV themselves, while 60 (35%) selected the middle category. Only 8 respondents (4%) reported frequently initiating HPV-related discussions. The mean score for physician-initiated HPV discussions was 2.38, with a median of 2.

Patient-initiated discussions about HPV were reported even less frequently. A total of 111 respondents (64%) indicated that patients rarely or very rarely initiate HPV-related conversations, 47 (27%) selected the middle category, and 15 respondents (9%) reported frequent patient initiation. The mean score for this item was 2.36, with a median of 2.

Parental initiation of HPV-related discussions occurred slightly more frequently than patient initiation. A total of 60 respondents (44%) reported rare or very rare parental initiation, 53 (39%) selected the middle category, and 23 respondents (17%) reported frequent parental initiation. The mean score for this item was 2.71, with a median of 3.

In a subset of consultations, HPV-related concerns represented the sole reason for seeking medical advice. This occurred frequently (scores 4–5) in 51 respondents (29%), while 81 (47%) reported this scenario as rare or very rare. The mean score for this item was 2.75, with a median of 3.

### 3.7. HPV Vaccination Offering Practices by Age Group and Sex

Patterns of HPV vaccination offering by patient age group and sex are presented in Table 8 (*n* = 136 respondents). HPV vaccination was most frequently offered to adolescent girls. A total of 80 respondents (59%) reported offering HPV vaccination often or very often (scores 4–5), while 18 respondents (13%) reported rarely offering vaccination (scores 1–2). The mean score for offering HPV vaccination to adolescent girls was 3.74, with a median of 4.

Offering HPV vaccination to adolescent boys showed a similar pattern. Frequent offering (scores 4–5) was reported by 78 respondents (57%), whereas 20 respondents (15%) indicated rare offering (scores 1–2). The mean score for this group was 3.70, with a median of 4.

HPV vaccination was offered less consistently to adult women. A total of 69 respondents (25%) reported frequent offering (scores 4–5), while 121 respondents (44%) selected rare offering (scores 1–2). The mean score for offering HPV vaccination to adult women was 2.80, with a median of 3.

Adult men represented the group with the lowest frequency of HPV vaccination offering. Only 23 respondents (9%) reported frequent offering (scores 4–5), whereas 194 respondents (70%) indicated that they rarely or very rarely offered HPV vaccination to adult male patients. The mean score for this item was 2.13, with a median of 2.

Overall, HPV vaccination was more frequently offered to female patients than to male patients across both adolescent and adult age groups, as reflected by higher mean and median scores for girls and women compared with boys and men. (Table 9.)

### 3.8. Association Between Knowledge Level and Participant Characteristics

The distribution of knowledge scores (bad, good, excellent) was compared across demographic and professional variables using chi-square tests.

A significant association was observed between knowledge level and sex (χ^2^ = 10.279, *p* = 0.006). Male participants showed a higher proportion of poor knowledge scores, whereas females more frequently achieved fair or excellent scores.

No statistically significant association was found between knowledge level and age group (χ^2^ = 15.89, *p* = 0.103), similarly, practice type (pediatric vs. adult) was not associated with knowledge category (χ^2^ = 2.28, *p* = 0.320).

Regarding practice location, a borderline but non-significant association was observed between urban versus rural practice and knowledge level (χ^2^ = 5.40, *p* = 0.067).

No significant relationship was identified between knowledge level and self-rated knowledge evaluation on a Likert scale (χ^2^ = 8.74, *p* = 0.365). Likewise, the association between knowledge level and belief in vaccine efficacy showed only a borderline trend toward significance (χ^2^ = 12.53, *p* = 0.051), suggesting a possible but inconclusive relationship between stronger belief in vaccine efficacy and higher knowledge scores (Table 10).

### 3.9. Factors Associated with HPV Vaccination Recommendation

Overall, 355 respondents (86.0%) reported that they regularly recommend HPV vaccination in their clinical practice, while 58 respondents (14.0%) reported that they do not. Associations between demographic characteristics, knowledge-related variables, and vaccination recommendation are summarized in Table 11.

Vaccination recommendation was strongly associated with self-rated HPV knowledge. Among physicians reporting good self-rated knowledge, 222 respondents recommended HPV vaccination and 17 did not, whereas among those reporting poor self-rated knowledge, 133 recommended vaccination and 41 did not. This association was statistically significant (χ^2^ = 22.57, *p* < 0.001). Physicians reporting good self-rated HPV knowledge had higher odds of recommending HPV vaccination compared with those reporting poor knowledge (OR = 4.03).

Composite HPV Knowledge Score categories were also associated with vaccination recommendation behavior (χ^2^ = 7.12, *p* = 0.028). Physicians with poor knowledge scores were less likely to recommend HPV vaccination compared with those with fair or excellent knowledge. Using the excellent knowledge category as the reference, the odds of not recommending HPV vaccination were higher among physicians with fair knowledge (OR = 1.60) and highest among those with poor knowledge (OR = 3.20).

Age showed a significant association with vaccination recommendation. Physicians older than 50 years were more likely to recommend HPV vaccination than those aged 50 years or younger (χ^2^ = 4.89, *p* = 0.027), corresponding to an odds ratio of 1.87 for recommending vaccination in the older age group.

Practice type was strongly associated with vaccination recommendation (χ^2^ = 28.17, *p* < 0.001). Pediatric general practitioners reported the highest rates of HPV vaccination recommendation, while adult general practitioners showed the lowest recommendation rates. When pediatric and adult practices were compared directly, pediatric GPs were substantially more likely to recommend HPV vaccination (OR = 16.06, *p* < 0.001).

Years spent in practice were also associated with vaccination recommendation in subgroup analyses. Physicians with less than 5 years of professional experience were less likely to recommend HPV vaccination compared with those with ≥30 years of experience (χ^2^ = 4.76, *p* = 0.029; OR = 0.36). In contrast, composite HPV Knowledge Scores and perceived vaccine effectiveness did not differ significantly between these two experience groups.

No significant associations were observed between vaccination recommendation and sex or geographic location (Budapest vs. non-Budapest).

## 4. Discussion

This nationwide cross-sectional survey provides the first comprehensive assessment of Hungarian general practitioners’ HPV-related knowledge, attitudes, communication comfort, and HPV vaccination recommendation practices across adult, pediatric, and mixed practices. Respondents demonstrated generally favorable perceptions of HPV vaccine safety and effectiveness and a high self-reported rate of regular recommendation, however clinically relevant knowledge gaps persisted, particularly around genotype–disease attribution and HPV-related malignancies. Sexual health communication was variably uncomfortable, routine STI/HPV conversations were infrequent and rarely initiated by physicians. Similarly to data available in the literature, vaccination recommendation behavior was strongly associated with provider self-rated knowledge, objective composite knowledge score, older age, and practice type, with pediatric GPs reporting markedly higher recommendation rates than adult practices [28,29,30,31,32,33,34].

### 4.1. Interpretation of HPV Knowledge and the “Confidence–Competence” Gap

Overall, participants correctly identified HPV as sexually transmitted and typically asymptomatic, and nearly all linked HPV to cervical cancer and genital warts. These findings suggest that the core narrative—HPV as a common STI and a necessary cause of cervical cancer—is well established among Hungarian primary care physicians, similarly to international examples [30,35,36]. However, the survey also detected nontrivial misconceptions. A meaningful proportion of respondents incorrectly attributed endometrial and ovarian cancers to HPV, and genotype-specific knowledge appeared inconsistent (e.g., a substantial minority associated HPV16/18 with genital warts and, conversely, selected low-risk types as causes of cervical cancer). Such patterns are consistent with international literature describing persistent provider knowledge gaps, particularly beyond cervical cancer and for genotype–phenotype relationships [33,37,38,39]. Importantly, nearly all physicians reported a need for further education, indicating receptivity to targeted professional development [24,30,37].

The association between self-rated knowledge and recommendation behavior (OR ≈ 4) is clinically consequential [40,41]. It suggests that perceived competence may influence vaccine advocacy as strongly as measured knowledge [42,43]. Furthermore, beliefs of the physicians themselves on vaccination do significantly influence recommendation confidence, which aligns with other studies [16,42,44,45,46].

This “confidence–competence” gap has practical implications: education programs should not only transmit factual content but also support counseling self-efficacy through structured communication training [18,19,34,47,48].

### 4.2. Recommendation Behavior: High Reported Rates, Yet Limited Routine Sexual Health Dialogue

A notable paradox in the findings is the coexistence of a high rate of self-reported regular HPV vaccination recommendation (86%) with the infrequent routine discussion of condom use, STIs, and HPV during consultations [49,50,51]. Although HPV-related topics were addressed more often than other aspects of sexual health, they were still reported to occur rarely and were seldom initiated by physicians [52,53]. This incongruity may partly reflect social desirability bias inherent to self-administered surveys; however, it more likely suggests that HPV vaccine “recommendation” is delivered in an opportunistic manner rather than being systematically embedded within comprehensive sexual health prevention counseling [7,36]. When vaccination advocacy remains episodic, opportunities for prevention are easily missed—particularly for adolescents outside structured immunization visits and for adult men, who were the least likely to be offered vaccination [13,54,55].

This pattern is consistent with the wider literature demonstrating that healthcare provider recommendation is the most influential determinant of HPV vaccine uptake and that the manner of recommendation is critical [18,19,28,41,52]. Presumptive, routine framing of HPV vaccination as standard care consistently outperforms participatory or hesitant approaches [19,56].

### 4.3. Barriers: Time Constraints Dominate; Financial Concerns Remain Salient

Among provider-level barriers, lack of time in everyday practice emerged as the most prominent obstacle, while “lack of data” about safety/efficacy and frequent guideline changes were generally rated as minor impediments. Similarly, a systematic review of global provider barriers found that, limited working time was the main obstacle to HPV vaccine recommendation, whereas concerns about safety and efficacy were less prominent in most countries [24]. This is an important distinction for intervention design. When knowledge deficits coexist with time constraints, conventional didactic education alone may have limited impact unless paired with workflow adaptations that reduce friction in busy clinics [40,57,58,59].

Cost was the most influential barrier attributed to patients (median 5, highest mean among patient-related factors), and financial concerns were also prominent for parents (especially outside the national program). This aligns with international observations that perceived or actual out-of-pocket costs and reimbursement uncertainty reduce both clinician recommendation and parental acceptance [58,60,61,62,63]. Furthermore, in our study we identified that doctors tend to underestimate the financial burden of the vaccination, that might lead to a negligent communication about participation in reimbursed vaccination program. For Hungary, these findings underscore the importance of clear communication to families regarding the possibility of free vaccination program at 13–14 years, as vaccination later on is not reimbursed, that might lead to vaccination hesitancy [61]. On the political level spreading coverage for latter ages could enhance vaccination frequency among adults [7,49,53].

### 4.4. Stigma and Communication Discomfort: A Persistent Upstream Determinant

Respondents perceived STI-related stigma as high, with HPV viewed as less stigmatizing yet still nontrivial, parallelly studies performed in Turkey or in the U.S. [64,65]. Communication comfort varied substantially by patient group and topic, and a sizable minority reported low comfort discussing sexuality with adolescents and in parent-present encounters. Notably, physicians generally rated discomfort as a limited direct barrier to recommendation frequency; however, discomfort may still exert an indirect effect by contributing to avoidance of broader sexual health discussions, narrowing counseling to technical vaccine details, or delaying conversations until late adolescence [18,66]. The finding that discussions about sexual health and condom use are rare suggests that stigma and discomfort likely shape clinic culture and conversation norms, even when clinicians do not identify them as primary barriers [64,67,68,69].

Given that parents were perceived as uncertain rather than strongly opposed—and that many were seen as uncomfortable discussing sexuality and inadequately informed—communication approaches that de-link vaccination from sexual activity may be particularly effective [66,70,71,72]. The American Academy of Pediatrics recommends normalizing HPV vaccination as standard preventive care and equipping clinicians to address common concerns (safety, necessity, and “too many vaccines”) while avoiding stigmatizing language [56]. Strong, presumptive recommendations and bundling HPV vaccine with other adolescent vaccines are evidence-based strategies to improve uptake [19,36,48,56]. Training should focus on these approaches to reduce stigma and improve vaccination rates

### 4.5. Determinants of Recommendation: Practice Type and Experience Effects

Practice type showed the strongest association with recommendation: pediatric GPs were substantially more likely to recommend HPV vaccination than adult practices. Pediatricians consistently report higher rates of strong HPV vaccine recommendations and use of evidence-based communication strategies compared to family physicians and adult medicine practitioners, a difference that is statistically significant and robust across international samples [20,73]. This likely reflects structural differences in workflow and patient contact patterns. Pediatric practices frequently deliver scheduled vaccines and may have established systems for counseling and documentation, while adult practices may perceive HPV vaccination as less central, encounter fewer routine adolescent preventive visits, and may be less exposed to immunization-related performance indicators [31,32]. The markedly lower offering to adult men further indicates that HPV vaccination remains framed as primarily female or adolescent-oriented, despite the recognized benefits for males and herd protection [13,36,74,75].

Interestingly, early-career physicians showed a lower likelihood of routinely recommending HPV vaccination. This finding should not be interpreted as reflecting insufficient knowledge, as recently trained practitioners are expected to have received up-to-date education regarding HPV prevention [16,41]. Rather, the difference may reflect factors not directly assessed in the present study, such as limited clinical experience, lower confidence in preventive counselling, organizational pressures during early professional development, or shorter established relationships with patients and parents [76,77]. Consequently, this result should be considered exploratory and warrants further investigation in studies specifically designed to assess behavioral and organizational determinants of vaccine recommendation practices [20,41,43].

### 4.6. Implications for Practice, Education, and Policy

Collectively, these results support a multipronged strategy to increase HPV vaccine uptake in Hungary. First, integration of targeted training on HPV prevention, vaccine communication, and implementation strategies into existing compulsory continuing medical education frameworks may represent a feasible strategy to improve knowledge translation into clinical practice, rather than relying exclusively on ad hoc voluntary educational initiatives [48]. These should address the specific misconceptions detected (HPV-related disease spectrum, genotype attribution, vaccination after HPV infection, and ongoing screening requirements), while also strengthening counseling self-efficacy [18,78].

Second, interventions must be designed for time-limited primary care settings: brief scripts, presumptive recommendation templates [19], electronic prompts [40], and delegation models (e.g., nurse-driven education) are likely to have higher impact than knowledge-based initiatives alone [59].

Third, financial barriers should be addressed with clear communication about who qualifies for publicly funded vaccination, together with transparent information on private-market costs, may help reduce perceived financial barriers [7,71]. Decisions regarding extension of reimbursement to additional age groups would require dedicated cost-effectiveness analyses and national policy evaluation [79].

Finally, adult practices should be prioritized for implementation support, given their comparatively lower recommendation rates, and particular attention should be paid to systematically including boys and adult men in counseling to avoid perpetuating gendered misconceptions about HPV prevention [12,74].

### 4.7. Strengths and Limitations

A major strength of this study is its national scope, inclusion of different practice types, and use of both objective and subjective measures, enabling triangulation between knowledge, confidence, and behavior. Collaboration with a patient advocacy group in questionnaire development is another strength, as it increases relevance to real-world counseling challenges.

Limitations include the restricted public availability of the email addresses of general practitioners presenting potential selection bias in the examined population.

Another limitation is the cross-sectional design, which precludes causal inference, and the modest response rate (17.1%), which may introduce selection bias toward physicians more engaged with preventive care.

Furthermore, female physicians, middle-aged practitioners, and doctors practicing in urban areas (particularly Budapest and Pest County) were overrepresented, however participant characteristics broadly overlapped with national workforce data. These differences likely reflect volunteer response patterns typical of physician surveys and should be considered when interpreting the generalizability of the findings.

All measures rely on self-report and may overestimate recommendation behaviors due to social desirability.

Lastly, although responses were obtained from all regions of Hungary, geographic coverage does not imply statistical representativeness, and the voluntary sampling approach may have resulted in differential participation across demographic and professional groups

## 5. Conclusions

Hungarian general practitioners exhibit solid awareness of HPV’s oncogenic potential and recognize vaccination as an essential preventive tool. However, incomplete understanding of vaccination schedules, gender inclusion, and counseling practices remains evident.

To strengthen the role of primary care in HPV prevention, enhanced continuing medical education, dissemination of up-to-date immunization guidelines, and communication skills training are recommended. GPs’ active engagement as trusted health communicators is essential in improving HPV vaccination coverage and reducing the burden of HPV-related malignancies.

Ultimately, empowering general practitioners to act as vaccination champions may accelerate progress toward the WHO cervical cancer elimination targets.

## Figures and Tables

**Table 1:** Basic characteristics of participants.

Variables	N (*n* = 413)	%	Reference Number [27]	Reference %
Age groups ^1^				
26–30	3	0.73	5328	12.36
31–40	58	14.04	9607	22.28
41–50	112	27.12	6712	15.56
51–60	116	28.09	7335	17.00
61–70	87	21.07	8344	19.35
70+	37	8.96	5795	13.44
Gender				
Female	298	72.15	3195	58.42
Male	115	27.85	2274	42.35
Years spent in practice				
0–4 years	56	13.56	NA	NA
5–9 years	56	13.56	NA	NA
10–19 years	109	26.39	NA	NA
20–29 years	92	22.28	NA	NA
30+ years	100	24.21	NA	NA
Practice location				
Bács-Kiskun county	16	3.87	297	5.43
Baranya county	17	4.12	248	4.53
Békés county	8	1.94	179	3.27
Borsod-Abaúj-Zemplén county	12	2.91	363	6.64
Budapest	142	34.62	1095	20.02
Csongrád-Csanád county	14	3.39	259	4.74
Fejér county	7	1.69	202	3.69
Győr-Moson-Sopron county	13	3.15	230	4.21
Hajdú-Bihar county	27	6.54	313	5.72
Heves county	4	0.97	155	2.83
Jász-Nagykun-Szolnok county	10	2.42	190	3.47
Komárom-Esztergom county	13	3.15	162	2.96
Nógrád county	0	0	95	1.74
Pest county	81	19.37	634	11.59
Somogy county	6	1.45	170	3.11
Szabolcs-Szatmár-Bereg county	9	2.18	300	5.49
Tolna	8	1.94	116	2.12
Vas	5	1.21	137	2.51
Veszprém	12	2.91	176	3.22
Zala county	9	2.18	148	2.71
Type of practice				
adult	240	58.11	4216	77.09
mixed	37	8.96		
pediatric	136	29.30	1253	22.91

^1^ Data refer to all doctors registered in Hungary. “NA” indicates that corresponding national reference data were not available for this variable.

**Table 2:** Basic knowledge of HPV and HPV screening.

Variables	N (*n* = 413)	%
What is the way of transmission for HPV?		
Sexually transmitted	389	94%
Hematogenic	28	7%
Droplets	21	5%
Direct contact	156	38%
Fecooral	29	7%
How prevalent do you think HPV infection is?		
Not at all	1	0%
Somewhat prevalent	21	5%
Prevalent	120	29%
Markedly prevalent	144	35%
Extremely prevalent	126	31%
In major part of patients does genital HPV infection cause symptoms?		
True	43	10%
False	368	89%
Which of the following are HPV related conditions?		
Genital warts	403	98%
Vulvar cancer	287	69%
Penile cancer	324	78%
Vaginal cancer	239	58%
Cervical cancer	408	99%
Endometrial cancer	82	20%
Ovarian cancer	45	11%
Anal cancer	275	67%
Malignant disease of the oral cavity	336	81%
Pharyngeal cancer	282	68%
What percentage of cervical cancer is related to HPV infection?		
50%	19	5%
60%	33	8%
70%	68	16%
80%	45	11%
90%	88	21%
more than 90%	160	39%
Which HPV genotypes can cause genital warts?		
6	329	80%
11	291	70%
16	124	30%
18	126	31%
31	83	20%
33	84	20%
45	74	18%
52	65	16%
58	60	15%
Which HPV genotypes can cause cervical cancer?		
6	340	82%
11	105	25%
16	320	77%
18	328	79%
31	165	40%
33	169	41%
45	155	38%
52	122	30%
58	125	30%
In which age groups is new HPV infection the most prevalent?		
0–10 years	1	0%
10–20 years	132	32%
20–30 years	320	77%
30–40 years	77	19%
40–50 years	14	3%
50–60 years	2	0%
60–70 years	3	1%
Who do you think, worth screening for HPV infection?		
Adolescent girls	168	41%
Adolescent boys	127	31%
Adult women	315	76%
Adult men	215	52%
Risk groups	281	68%
According to you, which screening method for cervical cancer is the most effective?		
Acetic acid-lugol solution staining and visualization	17	4%
Cytologic exam (Papanicolau test)	230	56%
HPV-PCR test	284	69%
HPV methylation test	25	6%
Biomarker test	30	7%
Do you think it is necessary to broaden your knowledge in this field?	115	27.85
Yes	391	95%
No	22	5%

**Table 3:** Basic knowledge of vaccination and attitudes towards vaccination of GPs.

Variables	N (*n* = 413)	%
How safe do you think HPV vaccination is?		
Not at all	0	0%
Little safe	0	0%
Somewhat safe	17	4%
Safe	90	22%
Very safe	306	74%
How effective do you think HPV vaccination is?		
Not at all	0	0%
Little effective	2	0%
Somewhat effective	30	7%
Effective	168	41%
Very effective	213	52%
Do you offer HPV vaccination regularly?		
Yes	355	86%
No	58	14%
For whom would you recommend HPV vaccination?		
Adolescent girls	392	95%
Adolescent boys	365	88%
Adult women	238	58%
Adult men	180	44%
Risk groups	276	67%
From which age is it advised to start HPV vaccination?		
9–12	178	43%
12–15	220	53%
15–18	15	4%
18–	0	0%
How many components does the currently available vaccine has in Hungary?		
2	20	5%
4	34	8%
9	354	86%
How much time, do you think, is needed to develop protection after receiving vaccination?		
1–2 days	0	0%
1–2 weeks	154	37%
1–2 months	231	56%
more than 12 months	28	7%
What do you think how much does a single vaccine cost?		
Mean	38,613 HUF	
Median	40,000 HUF	
In case of verified HPV infection which of the following are correct?		
Vaccination is not necessary as acquired immunity provides enough protection	4	1%
Vaccination is not necessary as further protection is not provided	15	4%
Vaccination is necessary as it protects from new infections	301	73%
Vaccination is necessary as it protects from re-infection	187	45%
I do not know	29	7%
Vaccination helps overcoming the already acquired infection.		
True	214	52%
False	197	48%
After HPV vaccination, cervix screening is not necessary anymore.	8	1.94
True	4	1%
False	407	99%
HPV vaccination can not be combined with other vaccinations.		
True	25	6%
False	386	93%
How many doses of vaccinations are needed for the whole schedule?	142	34.62
1	2	0%
2	30	7%
3	94	23%
2 or 3	287	69%

**Table 4:** Perceived professional role, knowledge adequacy, and stigma related to HPV and STIs.

How Much Do You Think…	1 Not at All		2		3		4		5 Totally		Average	Median
your knowledge about HPV is up-to-date?	10	(2%)	41	(10%)	196	(47%)	144	(35%)	22	(5%)	3.31	3
it is your task to talk about STIs?	4	(1%)	44	(11%)	131	(32%)	111	(27%)	122	(30%)	3.74	4
it is your task to talk about HPV?	5	(1%)	22	(5%)	100	(24%)	139	(34%)	147	(36%)	3.97	4
an STI stigmatizes?	9	(2%)	18	(4%)	77	(19%)	133	(32%)	175	(42%)	4.08	4
HPV infection stigmatizes?	41	(10%)	56	(14%)	150	(36%)	112	(27%)	54	(13%)	3.20	3

**Table 5:** Communication challenges.

How Comfortably Can You Communicate in the Following Situations?	1 Not at All	2	3	4	5 Totally	Average	Median
Talking about sexuality with adolescent boys	19 (11%)	34 (20%)	56 (32%)	39 (23%)	25 (14%)	2.19	2
Talking about sexuality with adolescent girls	10 (6%)	31 (18%)	51 (29%)	43 (25%)	38 (22%)	1.91	2
Talking about sexuality with parents	8 (5%)	29 (17%)	55 (32%)	45 (26%)	36 (21%)	1.95	2
Talking about sexuality with pediatrics patients in front of parents	16 (9%)	35 (20%)	52 (30%)	41 (24%)	29 (17%)	2.06	2
Talking about sexuality with adult men	22 (8%)	39 (14%)	70 (25%)	77 (28%)	69 (25%)	1.99	2
Talking about sexuality with adult women	11 (4%)	20 (7%)	68 (25%)	90 (32%)	88 (32%)	2.39	2
Talking about sexuality with non- heterosexual patients	35 (13%)	34 (12%)	80 (29%)	65 (23%)	63 (23%)	3.34	3
How confident are you talking about HPV?	9 (2%)	23 (6%)	102 (25%)	159 (38%)	120 (29%)	2.48	2

**Table 6:** What does influence the vaccination recommendation frequency?

How Does the Following Affects on Your Vaccination Suggestion Frequency?	1 Not at All	2	3	4	5 Totally	Average	Median
I feel uncomfortable to communicate about sexuality	168 (41%)	85 (21%)	93 (23%)	46 (11%)	(5%)	2.19	2
I feel uncomfortable to communicate about HPV	205 (50%)	91 (22%)	77 (19%)	30 (7%)	(2%)	1.91	2
I think vaccination guidance changes too often	185 (45%)	99 (24%)	100 (24%)	24 (6%)	(1%)	1.95	2
I am lacking data regarding vaccination efficacy	170 (41%)	102 (25%)	101 (24%)	28 (7%)	(3%)	2.06	2
I am lacking data regarding vaccination safety	184 (45%)	100 (24%)	87 (21%)	33 (8%)	(2%)	1.99	2
Parents and children get lot of information from several other sources	119 (29%)	103 (25%)	127 (31%)	40 (10%)	(6%)	2.39	2
There is not enough time for this in everyday practice	46 (11%)	64 (15%)	114 (28%)	80 (19%)	(26%)	3.34	3
I do not feel well-informed enough to open up the topic responsibly	113 (27%)	102 (25%)	117 (28%)	50 (12%)	(8%)	2.48	2

**Table 7:** Patients’ opinions about HPV and vaccination according to the experiences of doctors.

According to Your Experiences Are the Following Statements Valid?	1 Not at All	2	3	4	5 Totally	Average	Median
Patients have a negative attitude towards HPV vaccination	53 (19%)	67 (24%)	126 (45%)	25 (9%)	6 (2%)	2.51	3
Patients feel uncomfortable when they have to talk about sexuality	11 (4%)	44 (16%)	95 (34%)	85 (31%)	42 (15%)	3.37	3
Patients are not interested in non-obligatory vaccinations	20 (7%)	56 (20%)	101 (36%)	75 (27%)	25 (9%)	3.10	3
Patients do not believe in the efficacy of the HPV vaccination	28 (10%)	81 (29%)	124 (45%)	35 13(%)	9 (3%)	2.70	3
Patients do not believe in the safety of the HPV vaccination	30 (11%)	77 (28%)	110 (40%)	51 18(%)	9 (3%)	2.75	3
Patients do not think they might be in risk	12 (4%)	34 (12%)	102 (37%)	99 36(%)	30 (11%)	3.36	3
Patients think that HPV vaccination is not necessary beside condom use	83 (20%)	80 (19%)	155 (38%)	64 15(%)	31 (8%)	2.63	3
Patients think that HPV vaccination is too expensive	5 (2%)	11 (4%)	53 (19%)	68 25(%)	140 (51%)	4.18	5
Patients think they are informed enough about HPV	50 (18%)	106 (38%)	97 (35%)	18 6(%)	6 (2%)	2.36	2
Patients think they are informed enough about STIs	36 (13%)	82 (30%)	115 (42%)	38 (14%)	6 (2%)	2.62	3
Patients need further information about HPV	10 (4%)	47 (17%)	72 (26%)	74 (27%)	74 (27%)	3.56	4
Parents have a negative attitude towards the HPV vaccination of their children	32 (18%)	77 (45%)	45 (26%)	17 (10%)	2 (1%)	2.31	2
Parents feel uncomfortable when they have to talk about their children’s sexual activity	11 (6%)	37 (21%)	59 (34%)	47 (27%)	19 (11%)	3.15	3
Parents are well-informed about their children’s sexuality	29 (17%)	77 (45%)	50 (29%)	17 (10%)	0 (0%)	2.32	2
Parents speak about sexuality with their children at home	18 (10%)	74 (43%)	70 (40%)	11 (6%)	0 (0%)	2.43	2
Parents speak about STIs with their children at home	38 (22%)	83 (48%)	49 (28%)	3 (2%)	0 (0%)	2.10	2
Parents think they have to bring up the topic of sexuality at the age of 9–12	39 (23%)	78 (45%)	42 (24%)	11 (6%)	3 (2%)	2.20	2
Parents think they have to bring up the topic of sexuality at the age of 12–14	13 (8%)	42 (24%)	74 (43%)	37 (21%)	7 (4%)	2.90	3
Parents think they have to bring up the topic of sexuality at the age of 14–16	20 (12%)	30 (17%)	69 (40%)	43 (25%)	11 (6%)	2.97	3
Parents think they have to bring up the topic of sexuality at the age of 16–18	50 (29%)	36 (21%)	42 (24%)	35 (20%)	10 (6%)	2.53	3
Parents worry about the false sense of security towards STIs provided by vaccine	51 (29%)	53 (31%)	41 (24%)	21 (12%)	7 (4%)	2.31	2
Parents are less aware of non-obligatory vaccinations	18 (10%)	44 (25%)	52 (30%)	38 (22%)	21 (12%)	3.00	3
Parents think their children receive already too much vaccination	12 (7%)	38 (22%)	56 (32%)	46 (27%)	21 (12%)	3.15	3
Parents do not believe in the efficacy of the HPV vaccination	24 (14%)	59 (34%)	71 (41%)	11 (6%)	8 (5%)	2.54	3
Parents do not believe in the safety of the HPV vaccination	27 (16%)	55 (32%)	66 (38%)	16 (9%)	9 (5%)	2.57	3
Parents do not think that their children are at risk	14 (8%)	52 (30%)	61 (35%)	34 (20%)	12 (7%)	2.87	3
Parents think that outside the national program, vaccination is too expensive	11 (6%)	28 (16%)	46 (27%)	47 (27%)	41 (24%)	3.46	4
Parents think they are well-informed enough about HPV	18 (10%)	66 (38%)	70 (40%)	16 (9%)	3 (2%)	2.54	3
Parents think they are well-informed enough about STIs	21 (12%)	62 (36%)	68 (39%)	17 (10%)	5 (3%)	2.55	3
Parents think they need more information about HPV	8 (5%)	33 (19%)	52 (30%)	44 (25%)	36 (21%)	3.39	3
How informed do you think the patients are in this topic	23 (13%)	54 (31%)	81 (47%)	14 (8%)	1 (1%)	2.51	3
How informed do you think the parents are in this topic	11 (8%)	39 (29%)	69 (51%)	16 12(%)	1 (1%)	2.68	3

**Table 8:** Trends in consultation topics during GP visits.

How Often…	1 Not at All		2		3		4		5 Totally		Average	Median
do you speak about the topic of condom use during your consultations?	80	(19%)	205	(50%)	92	(22%)	27	(7%)	9	(2%)	2.22	2
do you speak about the topic of STIs during your consultations?	13	(8%)	90	(52%)	52	(30%)	4	(2%)	2	(1%)	2.23	2
do you speak about the topic of HPV and related pathologies during your consultations?	10	(6%)	79	(46%)	72	(42%)	10	(6%)	2	(1%)	2.51	2
do you open up the topic of HPV during your consultations?	13	(8%)	92	(53%)	60	(35%)	6	(3%)	2	(1%)	2.38	2
do your patients open up the topic of HPV during your consultations?	18	(10%)	93	(54%)	47	(27%)	12	(7%)	3	(2%)	2.36	2
do parents of your patients open up the topic of HPV during your consultations?	7	(5%)	53	(39%)	53	(39%)	19	(14%)	4	(3%)	2.71	3
does it occur that HPV is the only reason for asking help?	34	(20%)	47	(27%)	41	(24%)	30	(17%)	21	(12%)	2.75	3
do you talk about sexual health with your patients?	20	(12%)	102	(59%)	39	(23%)	12	(7%)	0	(0%)	2.25	2

**Table 9:** Trends in vaccination recommendation during consultations.

How Often…	1 Not at All		2		3		4		5 Totally		Average	Median
do you offer HPV vaccination for adolescent girls?	4	(3%)	14	(10%)	38	(28%)	37	(27%)	43	(32%)	3.74	4
do you offer HPV vaccination for adolescent boys?	4	(3%)	16	(12%)	38	(28%)	37	(27%)	41	(30%)	3.70	4
do you offer HPV vaccination for adult women?	21	(8%)	100	(36%)	87	(31%)	52	(19%)	17	(6%)	2.80	3
do you offer HPV vaccination for adult men?	76	(27%)	118	(43%)	60	(22%)	16	(6%)	7	(3%)	2.13	2

**Table 10:** Knowledge level and participant characteristics.

Variable	Poor	Fair	Excellent	χ^2^	*p*
Sex					
Male	14	65	36	10.279	0.006
Female	12	168	118	—	—
Age group					
26–30	0	2	1	15.89	0.103
31–40	3	40	15	—	—
41–50	6	59	47	—	—
51–60	6	65	45	—	—
61–70	4	49	34	—	—
70+	7	18	12	—	—
Practice type				—	—
Pediatric	16	140	84	2.28	0.320
Adult	5	66	50	—	—
Urban versus rural					
Budapest	14	81	47	5.40	0.067
Rural	12	152	107	—	—
Knowledge evaluation (Likert scale 1–5)					
1	1	3	0	8.74	0.365
2	1	12	5	—	—
3	10	82	60	—	—
4	10	117	74	—	—
5	4	19	15	—	—
Belief in vaccine efficacy (Likert scale 1–5)					
2	0	2	0	12.53	0.051
3	1	25	4	—	—
4	13	93	62	—	—
5	12	113	88	—	—

“—" indicates that the statistic was not applicable or not calculated for the respective category.

**Table 11:** Variables determining HPV vaccination offering.

Variable	Offers	Not Offers	χ^2^	*p*	OR	OR *p*
Knowledge						
Good	222	17	22.57	<0.001	—	—
Poor	133	41	—	—	4.03	<0.001
Sex						
Male	96	19	0.81	0.368	—	—
Female	259	39	—	—	0.76	0.369
Age group						
0–50 years	141	32	4.89	0.027	—	—
51–100 years	214	26	—	—	0.54	0.029
Region (East/West Hungary)						
West	76	14	0.22	0.641	—	—
East	279	44	—	—	0.86	0.641
Region (West/Central/East)						
West	76	14	0.26	0.876	—	—
Central	192	31	—	—	0.88	0.706
East	87	13	—	—	0.81	0.615
Years working as GP						
0–4 years	44	12	6.43	0.169	—	—
5–9 years	45	11	—	—	0.90	0.815
10–19 years	94	15	—	—	0.58	0.211
20–29 years	81	11	—	—	0.50	0.128
30+ years	91	9	—	—	0.36	0.034
Practice type						
Pediatric	134	2	25.02	<0.001	—	—
Adult ^1^	221	56	—	—	16.98	<0.001
Belief in HPV vaccine effectiveness						
Believes effective	336	45	20.30	<0.001	—	—
Does not believe	19	13	—	—	5.11	<0.001
Urban versus rural						
Budapest	124	18	0.34	0.563	—	—
Rural	231	40	—	—	1.19	0.563
Score evaluation						
Poor	126	31	7.12	0.028	—	—
Good	203	25	—	—	0.50	0.018
Excellent	26	2	—	—	0.31	0.126

^1^ Mixed practices were included in the adult category for statistical analyses, because of low numbers and their primarily adult-oriented patient population. “—" indicates that the statistic was not applicable or not calculated for the respective category.

## Data Availability

Anonymized data from this study are available upon reasonable request to support reproducibility.

## Supplementary Materials

The following supporting information can be downloaded at: https://www.mdpi.com/article/10.3390/vaccines14020196/s1, Table S1: HPV vaccination coverage in Europe (last dose) in 2024 [9].

## Abbreviations

The following abbreviations are used in this manuscript:

CME	Continuing medical education
GP	General practitioner
HPV	Human papillomavirus
IRB	Institutional Review Board
NEAK	National Health Insurance Fund Administration (Hungary)
NGO	Non-Government Organization
STI	Sexually Transmitted Infection
WHO	World Health Organization
