# Supplementary material for: Knowledge, Attitudes, and Practices of Hungarian General Practitioners Regarding Human Papillomavirus (HPV) Infection and Vaccination: A Nationwide Cross-Sectional Study
Source: Vaccines (Basel). 2026 Feb 22;14(2):196. doi: 10.3390/vaccines14020196 (PMC12944967; doi:10.3390/vaccines14020196)
Supplement: Supplementary file 1 [file vaccines-14-00196-s001.zip › vaccines-4140228-supplementary.pdf]

vaccines

Knowledge, Attitudes, and Practices of Hungarian General Practitioners Regarding Human Papillomavirus (HPV) Infection and Vaccination: A Nationwide Cross-Sectional Study

Richárd Tóth[1*], Pal Sebok[1], Eszter Börzsönyi[1], Icó Tóth[2], Barbara Sebők[3], Balázs Vida[1], Ferenc Bánhidy[1], Márton Keszthelyi[1$], Balázs Lintner[1$]

[1] Department of Obstetrics and Gynecology, Semmelweis University, 1082 Budapest, Hungary
[2] Mallow Flower Foundation, 1111 Budapest, Hungary
[3] Workgroup of Research Management, Doctoral School, Semmelweis University, 1085 Budapest, Hungary
$ These authors contributed equally to this work and are considered as last authors.
* Correspondence: toth.richard@semmelweis.hu

1. HPV Vaccination Questionnaire – Adult Practice

Respondent General Information

Sex:

- Female
- Male

Age:

- 26–30
- 31–40
- 41–50
- 51–60
- 61–70
- 70+

Practice location:

- Bács-Kiskun County
- Baranya County
- Békés County
- Borsod-Abaúj-Zemplén County
- Csongrád-Csanád County
- Fejér County
- Győr-Moson-Sopron County
- Hajdú-Bihar County
- Heves County
- Jász-Nagykun-Szolnok County
- Komárom-Esztergom County
- Nógrád County
- Pest County
- Somogy County
- Szabolcs-Szatmár-Bereg County

 https://doi.org/10.3390/xxxxx

- Tolna County
- Vas County
- Veszprém County
- Zala County
- Budapest

How long have you been working as a GP/family physician?

- 0–5 years
- 5–10 years
- 10–20 years
- 20–30 years
- 30+ years

Population size of the settlement where your practice is located: (free text)

Knowledge Related to HPV

Overall, how would you rate your knowledge about HPV? (1–5 scale)

Do you consider it necessary to expand your knowledge on this topic in your daily work?

- Yes
- No
- Maybe

From what sources would you expand your knowledge?

- Information materials provided by pharmaceutical companies
- Information materials provided by patient-support non-profit organizations
- Professional journals
- In-person continuing medical education
- Online continuing medical education
- I do not consider it necessary
- Other: ______

From which specialists would you be willing to accept training on this topic?

- Gynecologist
- Pediatrician / family physician
- Internist
- Microbiologist
- Epidemiologist
- Other: ______

What type of infection is HPV?

- Sexually transmitted
- Blood-borne
- Droplet transmission
- Direct contact
- Fecal–oral

How common is HPV infection? (1–5)

In most people, genital HPV infection causes symptoms.

- True
- False

Which conditions can be caused by HPV?

- Genital warts
- Vulvar cancer
- Penile cancer
- Vaginal cancer
- Cervical cancer
- Endometrial cancer
- Ovarian cancer
- Anal cancer
- Oral cancer
- Pharyngeal cancer

What percentage of cervical cancers is attributable to HPV infection?

- 50%
- 60%
- 70%
- 80%
- 90%
- More than 90%

Which HPV types can cause genital warts?

- 6
- 11
- 16
- 18
- 31
- 33
- 45
- 52
- 58

Which HPV types can cause cervical cancer?

- 6
- 11
- 16
- 18
- 31
- 33
- 45
- 52
- 58

In which age groups is newly acquired HPV infection most common?

- 0–10
- 10–20
- 20–30
- 30–40
- 40–50

- 50–60
- 60–70

Who should be screened for HPV infection?

- Adolescent girls
- Adolescent boys
- Adult women
- Adult men
- Individuals in high-risk groups

Which screening method do you consider the most effective for cervical cancer screening?

- Acetic acid–Lugol staining and visual inspection
- Cytology (Papanicolaou test)
- HPV PCR testing
- HPV methylation testing
- Biomarker testing

Knowledge Related to HPV Vaccination

Overall, how would you rate your knowledge about HPV vaccination? (1–5)

Do you consider it necessary to expand your knowledge on this topic in your daily work?

- Yes
- No

How safe do you consider HPV vaccination? (1–5)

If you do not consider it safe, why? (free text)

How effective do you consider HPV vaccination? (1–5)

If you do not consider it effective, why? (free text)

Do you regularly recommend HPV vaccination?

- Yes
- No

To whom would you recommend the vaccine?

- Adolescent girls
- Adolescent boys
- Adult women
- Adult men
- Individuals in high-risk groups
- I would not recommend it
- Other: ______

From what age is HPV vaccination recommended?

- From 9–12 years
- From 12–15 years
- From 15–18 years
- Above 18 years

How many components does the currently available vaccine in Hungary contain?

- 2
- 4
- 9

How long after vaccination does protection develop?
- 1–2 days
- 1–2 weeks
- 1–2 months
- More than 12 months

Approximately how much (in HUF) do you think one dose of HPV vaccine costs? (free text)

In the case of confirmed HPV infection:
- Vaccination is not necessary because acquired immunity provides protection
- Vaccination is not necessary because no beneficial effect can be expected
- Vaccination is necessary because it protects against new infections
- Vaccination is necessary because it protects against reinfection
- I do not know

Which of the following statements are true? (True/False)
- HPV vaccination helps eliminate an already established infection
- Cervical screening is no longer necessary after HPV vaccination
- HPV vaccination cannot be administered together with other vaccines

How many doses are required for the full vaccination series?
- 1
- 2
- 3
- 2 or 3 (depending on age)

In your opinion, to what extent does a confirmed STD infection stigmatize a person? (1–5)

In your opinion, to what extent does a confirmed HPV infection stigmatize a person? (1–5)

Facilitators and Barriers Related to Vaccination

Among your female patients to whom you recommended the vaccine, has anyone refused it?
- Yes
- No

Why did your female patients refuse it?
- Fear of potential side effects
- Do not believe in the vaccine's effectiveness
- Do not know the disease
- Because of the price
- Other: ______

Among your male patients to whom you recommended the vaccine, has anyone refused it?
- Yes
- No

Why did your male patients refuse it?
- Fear of potential side effects
- Do not believe in the vaccine's effectiveness
- Do not know the disease
- Because of the price
- Other: ______

To what extent do you consider it your own task to provide information about STDs? (1–5)

To what extent do you consider it your own task to provide information about HPV / HPV vaccination? (1–5)

On a scale from 1 to 5, how would you rate the following?

- How often do you discuss condom use?
- How often do you recommend HPV vaccination to adult women?
- How often do you recommend HPV vaccination to adult men?

How comfortable do you feel if:

- you have to talk about sexuality with adult men?
- you have to talk about sexuality with adult women?
- you have to talk about sexuality with a non-heterosexual patient?

How confident do you feel when discussing HPV with your patients? (1–5)

Based on your experience in your own practice, how true are the following statements? (1–5)

- Your patients have a negative attitude toward HPV vaccination
- Your patients feel uncomfortable when sexuality is discussed
- Your patients are not interested in non-mandatory vaccinations
- Your patients do not believe in the effectiveness of HPV vaccination
- Your patients do not believe in the safety of HPV vaccination
- Your patients do not think they may be at risk of such a threat
- Your patients believe that condom use is not necessary in addition to vaccination
- Your patients think the vaccine is too expensive
- Your patients think they have adequate knowledge about HPV
- Your patients think they have adequate knowledge about sexually transmitted infections
- Your patients would need more information about HPV

To what extent do you think your HPV vaccination recommendation habits are influenced by the following? (1–5)

- Communicating about sexuality is uncomfortable
- Communicating about HPV is uncomfortable
- Vaccination recommendations change too often
- You feel there are insufficient data on vaccine effectiveness
- You feel there are insufficient data on vaccine safety
- Parents and children receive information from many different sources on this topic
- There is not enough time in everyday practice to inform patients and parents
- You feel you are not sufficiently informed to discuss this responsibly

<u>2. HPV Vaccination Questionnaire – Pediatric Practice</u>

Respondent General Information

Sex:

- Female
- Male

Age:

- 26–30
- 31–40
- 41–50
- 51–60
- 61–70
- 70+

Practice location:

- Bács-Kiskun County
- Baranya County
- Békés County
- Borsod-Abaúj-Zemplén County
- Csongrád-Csanád County
- Fejér County
- Győr-Moson-Sopron County
- Hajdú-Bihar County
- Heves County
- Jász-Nagykun-Szolnok County
- Komárom-Esztergom County
- Nógrád County
- Pest County
- Somogy County
- Szabolcs-Szatmár-Bereg County
- Tolna County
- Vas County
- Veszprém County
- Zala County
- Budapest

How long have you been working as a GP/family physician?

- 0–5 years
- 5–10 years
- 10–20 years
- 20–30 years
- 30+ years

Population size of the settlement where your practice is located: (free text)

Knowledge Related to HPV

Overall, how would you rate your HPV-related knowledge? (1–5 scale)

Do you consider it necessary to expand your knowledge on this topic in your daily work?

- Yes
- No
- Maybe

From what sources would you expand your knowledge?

- Information materials provided by pharmaceutical companies
- Information materials provided by patient-support non-profit organizations
- Professional journals
- In-person continuing professional education
- Online continuing professional education
- I do not consider it necessary
- Other: ______

From which specialists would you be willing to accept training?

- Gynecologist
- Pediatrician / family physician
- Internist
- Microbiologist
- Epidemiologist
- Other: ______

What type of infection is HPV?

- Sexually transmitted
- Blood-borne
- Droplet transmission
- Direct contact
- Fecal–oral

How common is HPV infection? (1–5)

In most people, genital HPV infection causes symptoms.

- True
- False

Which conditions can be caused by HPV?

- Genital warts
- Vulvar cancer
- Penile cancer
- Vaginal cancer
- Cervical cancer
- Endometrial cancer
- Ovarian cancer
- Anal cancer
- Oral cancer
- Pharyngeal cancer

What percentage of cervical cancers is attributable to HPV infection?

- 50%
- 60%
- 70%
- 80%

https://doi.org/10.3390/xxxxx

- 90%
- More than 90%

Which HPV types can cause genital warts?

- 6
- 11
- 16
- 18
- 31
- 33
- 45
- 52
- 58

Which HPV types can cause cervical cancer?

- 6
- 11
- 16
- 18
- 31
- 33
- 45
- 52
- 58

In which age groups is newly acquired HPV infection most common?

- 0–10
- 10–20
- 20–30
- 30–40
- 40–50
- 50–60
- 60–70

Who should be screened for HPV infection?

- Adolescent girls
- Adolescent boys
- Adult women
- Adult men
- Individuals in high-risk groups

Which screening method do you consider the most effective for cervical cancer screening?

- Acetic acid–Lugol staining and visual inspection
- Cytology (Papanicolaou test)
- HPV PCR testing
- HPV methylation testing
- Biomarker testing

Knowledge Related to HPV Vaccination

Overall, how would you rate your knowledge about HPV vaccination? (1–5)

Do you consider it necessary to expand your knowledge on this topic in your daily work?

- Yes
- No

How safe do you consider HPV vaccination? (1–5)

If you do not consider it safe, why? (free text)

How effective do you consider HPV vaccination? (1–5)

If you do not consider it effective, why? (free text)

Do you regularly recommend HPV vaccination?

- Yes
- No

To whom would you recommend the vaccine?

- Adolescent girls
- Adolescent boys
- Adult women
- Adult men
- Individuals in high-risk groups
- I would not recommend it
- Other: ______

From what age is HPV vaccination recommended?

- From 9–12 years
- From 12–15 years
- From 15–18 years
- Above 18 years

How many components does the currently available vaccine in Hungary contain?

- 2
- 4
- 9

How long after vaccination does protection develop?

- 1–2 days
- 1–2 weeks
- 1–2 months
- More than 12 months

Approximately how much (in HUF) do you think one dose of HPV vaccine costs? (free text)

In the case of confirmed HPV infection:

- Vaccination is not necessary because acquired immunity provides protection
- Vaccination is not necessary because no beneficial effect can be expected
- Vaccination is necessary because it protects against new infections
- Vaccination is necessary because it protects against reinfection
- I do not know

Which of the following statements are true? (True/False)

- HPV vaccination helps eliminate an already established infection
- Cervical screening is no longer necessary after HPV vaccination
- HPV vaccination cannot be administered together with other vaccines

How many doses are required for the full vaccination series?

- 1
- 2
- 3
- 2 or 3 (depending on age)

In your opinion, to what extent does a confirmed STD infection stigmatize a person? (1–5)

In your opinion, to what extent does a confirmed HPV infection stigmatize a person? (1–5)

Facilitators and Barriers Related to Vaccination

Among your adolescent female patients to whom you recommended the vaccine, has anyone refused it?

- Yes
- No

Why did your adolescent female patients refuse it?

- Fear of potential side effects
- Do not believe in the vaccine's effectiveness
- Do not know the disease
- Because of the price
- Other: ______

Among your adolescent male patients to whom you recommended the vaccine, has anyone refused it?

- Yes
- No

Why did your adolescent male patients refuse it?

- Fear of potential side effects
- Do not believe in the vaccine's effectiveness
- Do not know the disease
- Because of the price
- Other: ______

To what extent do you consider it your own task to provide information about STDs? (1–5)

To what extent do you consider it your own task to provide information about HPV / HPV vaccination? (1–5)

On a scale from 1 to 5, how would you rate the following?

- How often do you talk about human papillomavirus infection and HPV-related conditions during consultations?
- How often do you bring up HPV as a topic?
- How often do your patients ask you about HPV and vaccination?
- How often do parents of your patients ask you about HPV and vaccination?
- Has it occurred that someone contacted your practice only for this reason (visit, phone, letter/email) to seek help?
- How often do you talk with your patients about their sexual life?
- How often do you discuss condom use?
- How often do you recommend HPV vaccination to adolescent girls?
- How often do you recommend HPV vaccination to adolescent boys?

How comfortable do you feel if:

- you have to talk about sexuality with adolescent boys?
- you have to talk about sexuality with adolescent girls?

https://doi.org/10.3390/xxxxx

- you have to talk about sexuality with parents?
- you have to talk about sexuality in the presence of parents?

How confident do you feel when discussing HPV with your patients? (1–5)

Based on your experience in your own practice, how true are the following statements? (1–5)

- Parents have a negative attitude toward their child's HPV vaccination
- Parents feel uncomfortable when their child's sexuality is discussed
- Parents are informed about their child's sexual activity
- Parents talk with their child about sexuality at home
- Parents talk with their child about sexually transmitted infections at home
- Parents think this topic should be discussed at ages 9–12
- Parents think this topic should be discussed at ages 12–14
- Parents think this topic should be discussed at ages 14–16
- Parents think this topic should be discussed at ages 16–18
- Parents worry that a sense of security from vaccination may lead to more irresponsible sexual behavior
- Parents are less concerned with non-mandatory vaccinations
- Parents feel their children already receive too many vaccines
- Parents do not believe in the vaccine's effectiveness
- Parents do not believe in the vaccine's safety
- Parents do not think their child may be at risk
- After the vaccination program, parents think the vaccine is too expensive
- Parents think they have adequate knowledge about HPV
- Parents think they have adequate knowledge about sexually transmitted infections
- Parents would need more information about HPV

How informed do you think your patients are about this topic? (1–5)

How informed do you think parents are about this topic? (1–5)

To what extent do you think your HPV vaccination recommendation habits are influenced by the following? (1–5)

- Communicating about sexuality is uncomfortable
- Communicating about HPV is uncomfortable
- Vaccination recommendations change too often
- You feel there are insufficient data on vaccine effectiveness
- You feel there are insufficient data on vaccine safety
- Parents and children receive information from many different sources on this topic
- There is not enough time in everyday practice to inform patients and parents
- You feel you are not sufficiently informed to discuss this responsibly

3. HPV Vaccination Questionnaire – Mixed Practice

Respondent General Information

Sex:

- Female
- Male

Age:

- 26–30
- 31–40
- 41–50
- 51–60
- 61–70
- 70+

Practice location:

- Bács-Kiskun County
- Baranya County
- Békés County
- Borsod-Abaúj-Zemplén County
- Csongrád-Csanád County
- Fejér County
- Győr-Moson-Sopron County
- Hajdú-Bihar County
- Heves County
- Jász-Nagykun-Szolnok County
- Komárom-Esztergom County
- Nógrád County
- Pest County
- Somogy County
- Szabolcs-Szatmár-Bereg County
- Tolna County
- Vas County
- Veszprém County
- Zala County
- Budapest

How long have you been working as a GP/family physician?

- **0–5 years**
- **5–10 years**
- **10–20 years**
- **20–30 years**
- **30+ years**

Population size of the settlement where your practice is located: (free text)

Knowledge Related to HPV

Overall, how would you rate your HPV-related knowledge? (1–5 scale)

Do you consider it necessary to expand your knowledge on this topic in your daily work?

- Yes
- No
- Maybe

From what sources would you expand your knowledge?

- Information materials provided by pharmaceutical companies
- Information materials provided by patient-support non-profit organizations
- Professional journals
- In-person continuing professional education
- Online continuing professional education
- I do not consider it necessary
- Other: ______

From which specialists would you be willing to accept training?

- Gynecologist
- Pediatrician / family physician
- Internist
- Microbiologist
- Epidemiologist
- Other: ______

What type of infection is HPV?

- Sexually transmitted
- Blood-borne
- Droplet transmission
- Direct contact
- Fecal–oral

How common is HPV infection? (1–5)

In most people, genital HPV infection causes symptoms.

- True
- False

Which conditions can be caused by HPV?

- Genital warts
- Vulvar cancer
- Penile cancer
- Vaginal cancer
- Cervical cancer
- Endometrial cancer
- Ovarian cancer
- Anal cancer
- Oral cancer
- Pharyngeal cancer

What percentage of cervical cancers is attributable to HPV infection?

- 50%
- 60%
- 70%
- 80%

- 90%
- More than 90%

Which HPV types can cause genital warts?

- 6
- 11
- 16
- 18
- 31
- 33
- 45
- 52
- 58

Which HPV types can cause cervical cancer?

- 6
- 11
- 16
- 18
- 31
- 33
- 45
- 52
- 58

In which age groups is newly acquired HPV infection most common?

- 0–10
- 10–20
- 20–30
- 30–40
- 40–50
- 50–60
- 60–70

Who should be screened for HPV infection?

- Adolescent girls
- Adolescent boys
- Adult women
- Adult men
- Individuals in high-risk groups

Which screening method do you consider the most effective for cervical cancer screening?

- Acetic acid–Lugol staining and visual inspection
- Cytology (Papanicolaou test)
- HPV PCR testing
- HPV methylation testing
- Biomarker testing

Knowledge Related to HPV Vaccination

Overall, how would you rate your knowledge about HPV vaccination? (1–5)

Do you consider it necessary to expand your knowledge on this topic in your daily work?

- Yes
- No

How safe do you consider HPV vaccination? (1–5)

If you do not consider it safe, why? (free text)

How effective do you consider HPV vaccination? (1–5)

If you do not consider it effective, why? (free text)

Do you regularly recommend HPV vaccination?

- Yes
- No

To whom would you recommend the vaccine?

- Adolescent girls
- Adolescent boys
- Adult women
- Adult men
- Individuals in high-risk groups
- I would not recommend it
- Other: ______

From what age is HPV vaccination recommended?

- From 9–12 years
- From 12–15 years
- From 15–18 years
- Above 18 years

How many components does the currently available vaccine in Hungary contain?

- 2
- 4
- 9

How long after vaccination does protection develop?

- 1–2 days
- 1–2 weeks
- 1–2 months
- More than 12 months

For this type of vaccine, do you think a booster dose is needed years later?

- Yes
- No
- Other: ______

Approximately how much (in HUF) do you think one dose of HPV vaccine costs? (free text)

In the case of confirmed HPV infection:

- Vaccination is not necessary because acquired immunity provides protection
- Vaccination is not necessary because no beneficial effect can be expected
- Vaccination is necessary because it protects against new infections
- Vaccination is necessary because it protects against reinfection
- I do not know

Which of the following statements are true? (True/False)

- HPV vaccination helps eliminate an already established infection
- Cervical screening is no longer necessary after HPV vaccination
- HPV vaccination cannot be administered together with other vaccines

How many doses are required for the full vaccination series?

- 1
- 2
- 3
- 2 or 3 (depending on age)

In your opinion, to what extent does a confirmed STD infection stigmatize a person? (1–5)

In your opinion, to what extent does a confirmed HPV infection stigmatize a person? (1–5)

Facilitators and Barriers Related to Vaccination

Among your adolescent female patients to whom you recommended the vaccine, has anyone refused it?

- Yes
- No

Why did your adolescent female patients refuse it?

- Fear of potential side effects
- Do not believe in the vaccine's effectiveness
- Do not know the disease
- Because of the price
- Other: ______

Among your adolescent male patients to whom you recommended the vaccine, has anyone refused it?

- Yes
- No

Why did your adolescent male patients refuse it?

- Fear of potential side effects
- Do not believe in the vaccine's effectiveness
- Do not know the disease
- Because of the price
- Other: ______

To what extent do you consider it your own task to provide information about STDs? (1–5)

To what extent do you consider it your own task to provide information about HPV / HPV vaccination? (1–5)

On a scale from 1 to 5, how would you rate the following?

- How often do you talk about human papillomavirus infection and HPV-related conditions during consultations?
- How often do you bring up HPV as a topic?
- How often do your patients ask you about HPV and vaccination?
- Has it occurred that someone contacted your practice only for this reason (visit or phone, or requested help by letter/email)?
- How often do you talk with your patients about their sexual life?
- How often do you discuss condom use?
- How often do you recommend HPV vaccination to adult women?
- How often do you recommend HPV vaccination to adult men?

How comfortable do you feel if:

- you have to talk about sexuality with adolescent boys?
- you have to talk about sexuality with adolescent girls?
- you have to talk about sexuality with parents?
- you have to talk about sexuality in the presence of parents?
- you have to talk about sexuality with adult men?
- you have to talk about sexuality with adult women?
- you have to talk about sexuality with a non-heterosexual patient?

How confident do you feel when discussing HPV with your patients? (1–5)

Based on your experience in your own practice, how true are the following statements? (1–5)

- Your patients have a negative attitude toward their child’s HPV vaccination
- Your patients feel uncomfortable when sexuality is discussed
- Your patients are not interested in non-mandatory vaccinations
- Your patients worry that a sense of security from vaccination may lead to more irresponsible sexual behavior
- Your patients are less concerned with non-mandatory vaccinations
- Your patients do not believe in the vaccine’s effectiveness
- Your patients do not believe in the vaccine’s safety
- Your patients think the vaccine is too expensive
- Your patients do not think they may be at risk of such a threat
- Your patients believe that condom use is not necessary in addition to vaccination
- Your patients think they have adequate knowledge about HPV
- Your patients think they have adequate knowledge about sexually transmitted infections
- Your patients would need more information about HPV

Based on your experience in your own practice, how true are the following statements regarding the parents of your pediatric patients? (1–5)

- Parents have a negative attitude toward their child’s HPV vaccination
- Parents feel uncomfortable when their child’s sexuality is discussed
- Parents are informed about their child’s sexual activity
- Parents talk with their child about sexuality at home
- Parents talk with their child about sexually transmitted infections at home
- Parents think this topic should be discussed at ages 9–12
- Parents think this topic should be discussed at ages 12–14
- Parents think this topic should be discussed at ages 14–16
- Parents think this topic should be discussed at ages 16–18
- Parents worry that a sense of security from vaccination may lead to more irresponsible sexual behavior
- Parents are less concerned with non-mandatory vaccinations
- Parents feel their children already receive too many vaccines
- Parents do not believe in the vaccine’s effectiveness
- Parents do not believe in the vaccine’s safety
- Parents do not think their child may be at risk
- After the vaccination program, parents think the vaccine is too expensive
- Parents think they have adequate knowledge about HPV
- Parents think they have adequate knowledge about sexually transmitted infections
- Parents would need more information about HPV

https://doi.org/10.3390/xxxxx

How informed do you think your patients are about this topic? (1–5)

How informed do you think parents are about this topic? (1–5)

To what extent do you think your HPV vaccination recommendation habits are influenced by the following? (1–5)

- Communicating about sexuality is uncomfortable
- Communicating about HPV is uncomfortable
- Vaccination recommendations change too often
- You feel there are insufficient data on vaccine effectiveness
- You feel there are insufficient data on vaccine safety
- Parents and children receive information from many different sources on this topic
- There is not enough time in everyday practice to inform patients and parents
- You feel you are not sufficiently informed to discuss this responsibly

Supplementary Table S1. HPV vaccination coverage in Europe (last dose) in 2024[9]

Region	Female (%)	Males (%)
Portugal	92	91
Norway	91	90
Spain	90	83
Cyprus	89	70
Iceland	89	90
Sweden	87	82
Denmark	81	77
Malta	80	49
Czechia	75	54
Hungary	75	66
United Kingdom	75	70
Ireland	73	69
Belgium	72	65
Switzerland	70	59
Estonia	63	44
Netherlands	63	59
Finland	62	53
Lithuania	59	59
Germany	55	34
Croatia	53	37
Italy	51	45
France	45	25
Latvia	44	51
Slovenia	43	25
Europe (as WHO Region)	38	25
Austria	31	24
Slovakia	24	12
Romania	17	1
Poland	13	7
Bulgaria	9	NA
Greece	NA	NA

https://doi.org/10.3390/xxxxx
